# 507. Impact of COVID-19 variant prevalence on hospitalization rate among individuals receiving monoclonal antibodies within a community health-system

**DOI:** 10.1093/ofid/ofad500.576

**Published:** 2023-11-27

**Authors:** Gretchen S Arnoczy, Andrew Kessell, Felicia M Vielbaum, Brenda Brock, Rebecca Carter, Jan M Scholl

**Affiliations:** FirstHealth of the Carolinas, Pinehurst, North Carolina; FirstHealth Moore Regional Hospital, Pinehurst, NC; FirstHealth Montgomery Memorial Hospital, Troy, North Carolina; FirstHealth Montgomery Memorial Hospital, Troy, North Carolina; FirstHealth Montgomery Memorial Hopital, Troy, North Carolina; FirstHealth Montgomery Memorial Hospital, Troy, North Carolina

## Abstract

**Background:**

During the COVID-19 pandemic, multiple targeted monoclonal antibodies were used in high risk patients with early COVID-19 to prevent complications. Serving as the primary referral site for a large geographic region in central North Carolina, our rural community health-system administered monoclonal agents to COVID-19 positive patients from December 2020 to November 2022. During this time period three predominate variants emerged (alpha, delta and omicron).

Monoclonal Antibody Infusions at FirstHealth of the Carolinas, December 2020-November 2022

**Figure:**
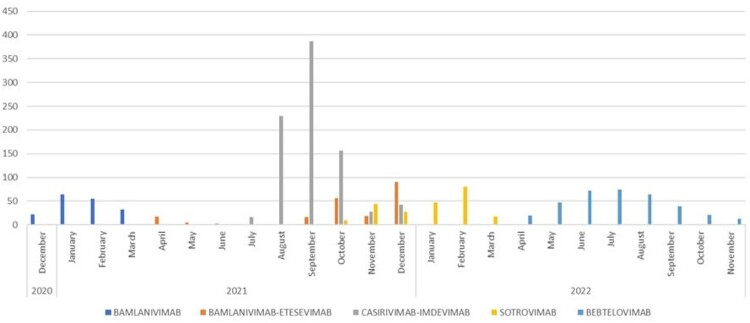

**Methods:**

A retrospective study was conducted at FirstHealth of the Carolinas Moore Regional Hospital examining patients from December 1^st^, 2020 to November 30^th^, 2022. All COVID-19 positive patients receiving one of the COVID-19 monoclonal therapies granted emergency use authorization during the study period were included. Patients were sub-divided into three distinct time periods representing different variants based on the prevalent pathogen in our region: December 2020 – June 2021 (alpha); July 2021- December 2021 (delta); January 2022-November 2022 (omicron). Hospitalization rates post monoclonal treatment were compared between time periods.

**Results:**

1820 monoclonal antibody infusions for COVID-19 were administered within the FirstHealth of the Carolinas system in the period studied. Number of infusions were highest during delta with over 60% of the infusions occurring during this period. Hospitalization rates in this group were 3.5% (alpha), 3.5% (delta), and 2.0% (omicron). Vaccination status among individuals getting monoclonal antibody infusions changed over time based on changing infusion criteria and variant prevalence.

**Conclusion:**

In a real world, rural, community health care setting in the Southeast United States - hospitalization rates for high-risk individuals receiving monoclonal antibody infusions for COVID-19 were similar to reported rates and varied by variant prevalence. This is may be due to alterations in variant severity as well as vaccination status of the population.

**Disclosures:**

**All Authors**: No reported disclosures

